# Sex dependent regulation of osteoblast response to implant surface properties by systemic hormones

**DOI:** 10.1186/2042-6410-1-4

**Published:** 2010-11-04

**Authors:** Rene Olivares-Navarrete, Sharon L Hyzy, Reyhaan A Chaudhri, Ge Zhao, Barbara D Boyan, Zvi Schwartz

**Affiliations:** 1 Institute for Bioengineering and Bioscience, Georgia Institute of Technology, 315 Ferst Drive, Atlanta, GA, USA

## Abstract

**Background:**

Osseointegration depends on the implant surface, bone quality and the local and systemic host environment, which can differ in male and female patients. This study was undertaken in order to determine if male and female cells respond differently to titanium surfaces that have micron-scale roughness and if interactions of calciotropic hormones [1α,25(OH)_2_D_3 _and 17β-oestradiol (E_2_)] and microstructured surfaces on osteoblasts are sex dependent.

**Methods:**

Osteoblasts from 6-week old Sprague-Dawley rats were cultured on tissue culture polystyrene (TCPS) or on titanium (Ti) disks with two different surface topographies, a smooth pretreated (PT) surface and a coarse grit-blasted/acid-etched (SLA) surface, and treated with 1α,25(OH)_2_D_3_, E_2_, or E_2 _conjugated to bovine serum albumin (E_2_-BSA).

**Results:**

Male and female cells responded similarly to Ti microstructure with respect to cell number and levels of osteocalcin, transforming growth factor-β1, osteoprotegerin and prostaglandin E_2 _in their conditioned media, exhibiting a more differentiated phenotype on SLA than on PT or TCPS. E_2 _and E_2_-BSA increased differentiation and local factor production, an effect that was microstructure dependent and found only in female osteoblasts. 1α,25(OH)_2_D_3 _increased osteoblast differentiation and local factor production in female and male cells, but the effect was more robust in male cells.

**Conclusions:**

Male and female rat osteoblasts respond similarly to surface microstructure but exhibit sexual dimorphism in substrate-dependent responses to systemic hormones. Oestrogen affected only female cells while 1α,25(OH)_2_D_3 _had a greater effect on male cells. These results suggest that successful osseointegration in males and females may depend on the implant surface design and correct levels of calciotropic hormones.

## Background

Orthopaedic diseases, such as osteoporosis, osteoarthritis and some spinal disorders, are more prevalent in women [1]. Until recently, most of these differences were attributed to the circulating levels of oestrogen and testosterone. However, there is increasing evidence that sexual dimorphism occurs before puberty and depends on fundamental differences at the genetic level. Bone properties, including size and shape, are inherited differently in males and females [2]. Components of the extracellular matrix (small integrin binding, N-linked glycoproteins [SIBLING]) are encoded on the X-chromosome, causing differential expression of resulting syndromes [3]. Sexual dimorphism also exists in lineage preference of mesenchymal stem cells (MSCs) [4,5].

An ageing population has increased demand for dental implants to replace missing teeth and orthopaedic implants to restore function. Successful osseointegration of these implants, defined as direct contact between bone and the implant surface under loading conditions [6,7], depends in part on bone quality and the local and systemic host environment. Studies have shown lower osseointegration rates in osteopenic bone than in normal bone [8]; in osteoporotic animals osseointegration is 50% slower than in normal experimental animals [9,10]. Moreover, clinical trials have shown that osteoporosis is significantly related to early implant failures in males and females [11] and that age is a major contributing factor [12].

Age certainly is an important variable, but systemic hormones also contribute to early implant failure. In oestrogen-deficient animals, osseointegration is impaired, and this represented by less bone mass, a smaller contact area between bone and the implant [13,14] and decreased pull-out strength of implants [15]. Similarly, postmenopausal women have had significantly more failures of maxillary implants than their premenopausal counterparts [16]. One possibility is that there is a biphasic effect of female gonadal hormone deficiency, which may temporarily interfere in early implant-tissue integration and may be associated with a failure to upregulate a select set of bone extracellular matrix genes [9].

Some studies suggest that oestrogen replacement therapy may promote bone healing around titanium (Ti) implants in osteoporotic bone [17,18]. However, other studies do not show improved implant success rates [12]. This raises the possibility that the design of the implant may also be a contributing factor. Female osteoblasts are sensitive to differences in surface morphology and their responses to oestrogen vary with the surface properties [19]. Oestrogen is also necessary for bone strength in males [20] and male osteoblasts are sensitive to differences in the surface properties of Ti and Ti alloy implants [21-23], but whether oestrogen regulates the response of male osteoblasts to orthopaedic implant materials is not known.

The vitamin D metabolite 1,25-dihydroxyvitamin D3 [1α,25(OH)_2_D_3_] is another important factor regulating osteoblast differentiation and bone formation. The prevalence of vitamin D deficiency is relatively higher in certain populations, such as the elderly [24], pregnant women in the northern USA [25] or people with limited sun exposure [26]. Recent reports indicate that fracture healing is reduced in vitamin D deficient women [27], suggesting that 1α,25(OH)_2_D_3 _may also modulate osteoblasts in a sex specific manner.

Successful osseointegration of an implant depends on its surface properties. *In vivo*, osteoblasts proliferate and differentiate in areas of bone conditioned by osteoclasts, leaving a micron-scale roughness [28]. Microstructured Ti and Ti-6Al-4V surfaces, with a roughness similar to that found in the resorption lacunae left by osteoclasts [29], result in higher bone-to-implant contact and stronger mechanical integration *in vivo *than smooth surfaces, resulting in better osseointegration [30-33]. This result is supported by *in vitro *findings showing that microstructured surfaces enhance osteoblast differentiation, matrix deposition and the production of osteogenic growth factors [21-23], which regulate the cells via autocrine and paracrine pathways [34-36]. Osteoblast response to exogenous regulators, including systemic osteotropic hormones, is also sensitive to surface properties. The effects of 1α,25(OH)_2_D_3 _on human osteoblast-like MG63 cells, normal human osteoblasts, fetal rat calvarial cells and mouse osteocyte-like cells are enhanced on rougher surfaces [37,38].

The aim of the present study was to determine if sexual dimorphism in osteoblasts involves a differential response to implant surface microstructure and if the interactions between the response to surface microstructure and the calciotropic hormones 1α,25(OH)_2_D_3 _and 17β-oestradiol are sex-dependent. The study was performed using primary osteoblasts isolated from adult male and female rats. However, it does not address the question of whether sexual dimorphism at the cell level affects the long-term success of orthopaedic implants *in vivo*.

## Methods

We used an *in vitro *model to determine whether male and female osteoblasts exhibit differential responses to Ti surface microstructure and if there are substrate dependent differences in response to systemic hormones. Primary rat osteoblasts were cultured to confluence on Ti disks with three different surface morphologies. At confluence, they were treated for 24 hours with oestrogen (17β-oestradiol) or with 1α, 25(OH)_2_D_3_. Outcome measures included cell number, alkaline phosphatase specific activity, osteocalcin production and the production of growth factors. In each experiment there were six independent cultures per variable (male, female, surface, hormone concentration) and all experiments were repeated to ensure the validity of the findings.

### Disk preparation

Ti disks with a 15 mm diameter were prepared from Ti sheets as described previously [29]. Pretreatment disks (PT) were cleaned by washing in acetone and processing them through a 2% ammonium fluoride/2% hydrofluoric acid/10% nitric acid solution at 55°C for 30 s. In order to produce disks with a mixed micron scale and submicron scale topography (coarse grit-blasted/acid-etched, SLA), PT disks were coarse grit-blasted with 0.25-0.50 mm corundum until the surface reached a uniform gray tone, followed by acid etching. The surface parameters for each of the surfaces have been described in detail [39]. The PT surface is comparatively smooth with an average peak to valley roughness (Ra) of 40 nm. The SLA surface is characterized by 30 to 100 μm craters overlaid with 1-3 μm pits, resulting in an Ra of 3.2 μm. Control cultures were grown on standard tissue culture polystyrene (TCPS).

### Cell culture

Osteoblasts were isolated from frontal and parietal (calvaria) bones of 6-week-old male (*n *= 8) and female (*n *= 8) Sprague-Dawley rats using an explant isolation technique [40]. Periosteum and soft tissues were removed from the bones. Pieces with a 1-2 mm diameter were washed three times in Hank's balanced salt solution (HBSS) containing penicillin-streptomycin, and digested for 15 minutes at 37°C with 0.25% trypsin-ethylenediaminetetraacetic acid (EDTA; Invitrogen, CA, USA). The digestion was discarded in order to avoid fibroblast contamination. The bone chips were placed in a 100 × 20 Petri dish (BD, NJ, USA) and cultured in Dulbecco's modified Eagle medium (DMEM; cellgro^®^, Mediatech, Inc, VA, USA) containing 1% penicillin-streptomycin (Invitrogen) and 10% fetal bovine serum (Hyclone, UT, USA). At confluence, the cells were subpassaged and cultured as above. Confluent third passage cultures were used for the experiments described below. In order to ensure that these cells retained osteoblastic responses to 1α,25(OH)_2_D_3 _at this passage, we examined the dose-dependent effects of the seco-steroid on cell number, alkaline phosphatase specific activity and osteocalcin production. Confluent cultures were treated with 10^-9^M or 10^-8^M 1α,25(OH)_2_D_3 _for 24 hours.

Validated rat osteoblasts were plated at 20,000 cells/cm^2 ^on TCPS, PT and SLA substrates. Media were exchanged at 24 hours and then every 48 hours until cells reached confluence on TCPS. At confluence, media were replaced with experimental media containing vehicle or 1α,25(OH)_2_D_3 _(10^-9 ^and 10^-8^M) (Biomol Research Laboratories, PA, USA) or 10^-9^M 17β-oestradiol (E_2_) or E_2 _conjugated to bovine serum albumin (E_2_-BSA, Sigma-Aldrich Co, MO, USA), which prevents diffusion of E_2 _across the plasma membrane, thereby activating membrane-mediated E_2_-signalling [41].

### Biochemical analysis

When the osteoblast cultures achieved confluence on TCPS, all the cultures were treated for 24 hours with media containing either vehicle or the appropriate hormone concentration. The cell number was determined at harvest for all the cultures. Cells were released by two sequential 10 minute incubations in 0.25% trypsin at 37°C in order to ensure that all cells were removed from rough Ti surfaces and counted (Z1 cell and particle counter, Beckman Coulter, CA, USA). In order to measure the cellular alkaline phosphatase specific activity [orthophosphoric monoester phosphohydrolase, alkaline; E.C. 3.1.3.1], cells were lysed by freeze-thawing in Triton-X100. Enzyme activity in the lysates was assayed by measuring the release of *p*-nitrophenol from *p*-nitrophenylphosphate at pH 10.2 and results were normalized to the protein content of the cell lysates. Osteocalcin in the conditioned media was measured using a commercially available radioimmunoassay kit (Human Osteocalcin RIA Kit, Biomedical Technologies, MA, USA) and normalized by cell number. The conditioned media were also assayed for growth factors and cytokines. The prostaglandin E_2 _(PGE_2_) was assessed using a commercially available competitive binding radioimmunoassay kit (Prostaglandin E_2 _RIA kit, Perkin Elmer, MA, USA). Active transforming growth factor (TGF)-β1 was measured prior to acidification of the conditioned media, using an enzyme-linked immunoassay (ELISA) kit specifically for human TGF-β1 (TGF-β1 DuoSet, R&D Systems, MN, USA). The total TGF-β1 was measured after acidifying the media and the latent TGF-β1 was defined as total TGF-β1 minus active TGF-β1. The osteoprotegerin (OPG) levels were measured using an ELISA kit (Osteoprotegerin DuoSet, R&D Systems).

### Statistical analysis

The experimental design was controlled as far as possible in order to ensure that any sex differences were not due to an artifact. Male or female cells were collected from a minimum of eight rats each for each experiment. Rats of the same sex were euthanized on the same day and cells were harvested together. Male and female cells were collected one week apart. In order to eliminate the possibility that differences between male and female cell behaviour were artifacts of different tissue culture conditions, we strictly controlled the culture conditions and used the same processes to sterilize the Ti surfaces, cell isolation procedures, cell seeding densities and culture media. Moreover, the biochemical analyses were performed at the same time.

Each variable was tested in six independent cultures for each experiment so that we could statistically compare the responses of the male and female cells to each surface and to each hormone treatment on a specific surface in a single experiment. All the experiments were repeated in order to ensure the validity of the results. The data presented are from representative experiments. The data were first analysed by analysis of variance; when statistical differences were detected, Student's *t*-test for multiple comparisons using Bonferroni's method was used. We compared the responses on each Ti surface to the response on TCPS for each hormone treatment. In addition, we compared the response to each dose of hormone to the response to control media for each of the surfaces.

## Results

### Phenotype characterization

**Figure 1:**
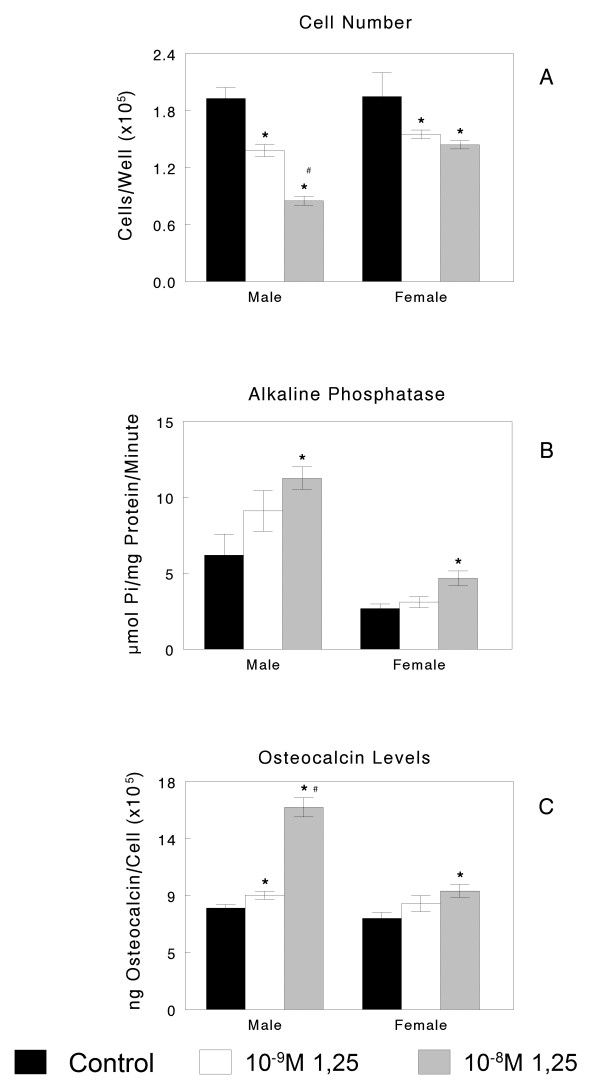
**Osteoblasts from female and male rats exhibit differential responses to 1α,25(OH)**_**2**_**D**_**3 **_**when cultured on tissue culture polystyrene**. Osteoblasts isolated from the calvaria of female and male rats were treated for 24 hours with increasing concentrations of the vitamin D metabolite 1α,25(OH)_2_D_3 _at confluence. Cell number (A), alkaline phosphatase specific activity in cell lysates (B) and osteocalcin in the conditioned media (C) were measured after 24 hours of treatment with either vehicle, 10^-9^M 1α,25(OH)_2_D_3 _or 10^-8^M 1α,25(OH)_2_D_3_. *P < 0.05, 1α,25(OH)_2_D_3 _treatment versus control; #*p *< 0.05, 10^-9^M 1α,25(OH)_2_D_3 _treatment versus 10^-8^M treatment.

Cells isolated from the calvaria of male and female rats exhibited characteristics typical of osteoblasts (Figure 1). Confluent cultures of third passage cells exhibited alkaline phosphatase activity, an early marker of osteoblast maturation, which reaches a peak just before matrix mineralization [42]. In addition, these cells produced osteocalcin, a later marker of osteoblast maturation, which plays and important part in modulating hydroxyapatite crystal formation [43]. When grown under control conditions on TCPS, there was no evidence of sexual dimorphism between female and male osteoblasts. Moreover, both female and male cells exhibited dose-dependent responses to 1α,25(OH)_2_D_3 _typical of osteoblasts, characterized by decreased cell number (Figure 1A), increased alkaline phosphatase specific activity (Figure 1B) and osteocalcin production (Figure 1C). Although the effect of 1α,25(OH)_2_D_3 _on alkaline phosphatase was similar in the male and female cells, the effects of 1α,25(OH)_2_D_3 _on cell number and osteocalcin were more robust in the male cells, indicating the response to hormonal regulation may have a sex-specific component.

### Cell response to Ti substrates

**Figure 2:**
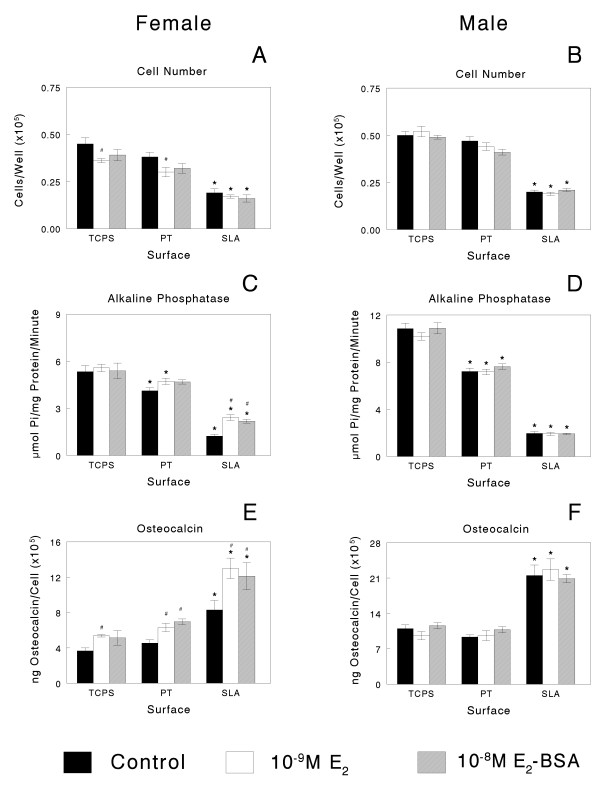
**Only female cells respond to oestrogen when cultured on smooth and microstructured titanium (Ti) substrates**. Osteoblasts from male and female rats were plated on Ti surfaces and treated with either E_2 _or E_2 _conjugated to bovine serum albumin (BSA; E_2_-BSA) at confluence. Cell number (A, B), alkaline phosphatase specific activity in cell lysates (C, D) and osteocalcin in the conditioned media (E, F) were measured after 24 hours. *P < 0.05, Ti surfaces versus tissue culture polystyrene; #*P *< 0.05, oestradiol versus control.

**Figure 3:**
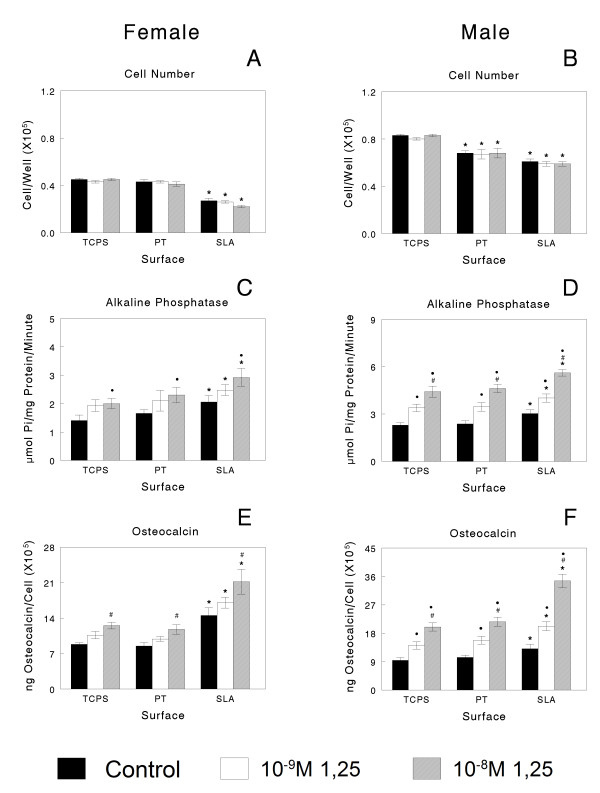
**Male osteoblasts respond more robustly to 1α,25(OH)**_**2**_**D**_**3 **_**than females, an effect enhanced on microstructured titanium (Ti)**. Confluent cultures of rat calvarial osteoblasts were plated on Ti surfaces and treated with 1α,25(OH)_2_D_3_. Cell number (A, B), alkaline phosphatase specific activity in cell lysates (C, D) and osteocalcin in the conditioned media (E, F) were measured after 24 hour treatment with either vehicle, 10^-9^M 1α,25(OH)_2_D_3 _or 10^-8^M 1α,25(OH)_2_D_3_. *P < 0.05, Ti surfaces versus tissue culture polystyrene at each concentration; #*P *< 0.05, 1α,25(OH)_2_D_3 _versus control; black circle, *P *< 0.05, 10^-9^M 1α,25(OH)_2_D_3 _versus 10^-8^M.

When female and male cells were cultured on Ti substrates under control conditions, there was no evidence of sexual dimorphism (Figures 2 and 3). In cultures treated with media containing the vehicle alone, female cell numbers were comparable on TCPS and PT but lower on SLA (Figure 2A); male cell numbers were reduced on PT and SLA in comparison with TCPS (Figure 2B). Alkaline phosphatase was reduced on PT and further reduced on SLA in comparison to TCPS, although osteocalcin levels were increased on SLA in both male and female cells, indicating a more differentiated phenotype [42]. In a second set of control cultures, alkaline phosphatase (Figure 3C and 3D) and osteocalcin (Figure 3E and 3F) were increased in male and female cells grown on SLA, indicating that the cells were harvested at an early state of differentiation due to small differences in confluence.

**Figure 4:**
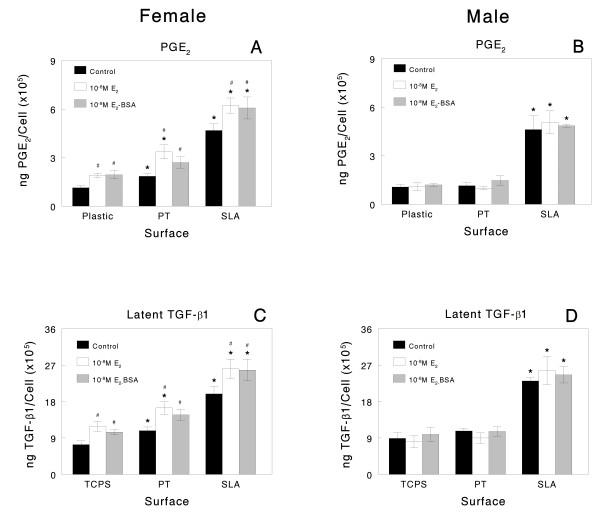
**Only female osteoblasts increase production of prostaglandin (PGE**_**2) **_**and transforming growth factor (TGF-β1) after oestrogen treatment when grown on titanium (Ti) surfaces**. Osteoblasts isolated from calvaria of male and female rats were plated on Ti surfaces. At confluence, cells were treated with E_2 _or E_2_-BSA for 24 hours. Levels of PGE_2 _(A, B) and latent TGF-β1(C, D) were measured in the conditioned media. *P < 0.05, Ti surfaces versus tissue culture polystyrene; #*P *< 0.05, oestradiol versus control.

**Figure 5:**
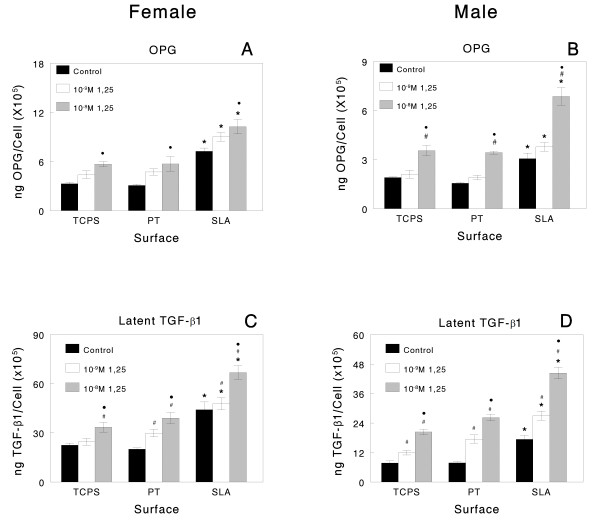
**Male osteoblasts respond more robustly to 1α,25(OH)**_**2**_**D**_**3 **_**than females, an effect enhanced on microstructured titanium (Ti)**. Osteoblasts isolated from calvaria of male and female rats were plated on Ti surfaces. At confluence, cells were treated with 1α,25(OH)_2_D_3 _for 24 hours. Levels of osteoprotegerin (A, B) and latent tissue culture polystyrene (TCPS)-β1 (C, D) were measured in the conditioned media. *P < 0.05, Ti surfaces versus TCPS; #*P *< 0.05, 1α,25(OH)_2_D_3 _versus control; **·***P *< 0.05, 10^-9^M 1α,25(OH)_2_D_3 _versus 10^-8^M.

Control cultures of both female and male osteoblasts exhibited substrate-dependent increases in the production of local factors. Female cells exhibited higher levels of PGE_2_, a cytokine that regulates osteoblast differentiation [44], in their conditioned media on both Ti substrates compared to TCPS (SLA > PT) whereas male osteoblasts only had increased PGE_2 _levels on SLA (Figure 4A and 4B). Females had higher levels of OPG, the RANK ligand decoy receptor important in decreasing osteoclastic activity, in their conditioned media than male cells, regardless of the surface, and exhibited a greater increase when grown on SLA in comparison to PT or TCPS (Figure 5A and 5B). Female and male cells exhibited comparable responses to surface microstructure with respect to the levels of latent TGF-β1 (Figures 4C and 4D and 5C and 5D) and active TGF-β1 (data not shown; SLA > TCPS), a growth factor that enhances extracellular matrix production.

### Osteoblast response to 17β-oestradiol

Sexual dimorphism was evident in the response of cells to oestrogen, with responses to the hormone being evident only in the cells from female rats (Figures 2 and 4). Moreover, the response of the female cells was substrate dependent. E_2 _caused a decrease in the number of cells in females on TCPS and PT but had no effect on cell numbers on the rough SLA surface (Figure 2A). In contrast, E_2 _had no effect on male osteoblast numbers on any substrate (Figure 2B). E_2_-BSA did not regulate cell numbers in either female or male cultures. Both forms of the hormone stimulated alkaline phosphatase in female osteoblasts but only in cultures grown on SLA (Figure 2C). Neither form of oestrogen affected the enzyme activity in male cells (Figure 2D). Female cells responded to E_2 _and E_2_-BSA with an increase in osteocalcin on all surfaces but the percent increase was greatest in cells grown on SLA (Figure 2E). Neither E_2 _nor E_2_-BSA affected osteocalcin in the male cultures (Figure 2F). E_2 _and E_2_-BSA increased PGE_2 _(Figure 4A) and latent TGF-β1 (Figure 4C) in the media of female cells grown on all substrates. Both forms of oestrogen increased the levels of active TGF-β1 (data not shown). Neither E_2 _nor E_2_-BSA affected the levels of PGE_2 _(Figure 4B) or of latent (Figure 4D) or active (data not shown) TGF-β1 in the media of male cells.

### Osteoblast response to 1α,25(OH)_2_D_3_

As noted above, both male and female cells grown on Ti responded to 1α,25(OH)_2_D_3 _but responses were more robust in the male cells, particularly on the microstructured SLA substrates (Figures 3 and 5). 1α,25(OH)_2_D_3 _had no effect on cell numbers in either female or male cultures grown on any of the test surfaces (Figure 3A and 3B). Alkaline phosphatase in female cells was increased by 10^-8^M 1α,25(OH)_2_D_3 _on all substrates (Figure 3C). Male cells were more sensitive to 1α,25(OH)_2_D_3_, showing increases at 10^-9^M (Figure 3D). 1α,25(OH)_2_D_3 _also affected osteocalcin production in both the male and female cells on all substrates. However, female cells exhibited increases only at the higher dose (Figure 3E). Moreover, male cells exhibited a synergistic stimulation in osteocalcin production when grown on SLA and treated with 10^-8^M 1α,25(OH)_2_D_3 _(Figure 3F). Female and male osteoblasts responded to 10^-8^M 1α,25(OH)_2_D_3 _with an increase in OPG but the effect was more pronounced in male cells grown on SLA and was synergistic with surface microstructure (Figure 5A and 5B). 1α,25(OH)_2_D_3 _also caused an increase in latent TGF-β1 levels on all surfaces, with the greatest stimulatory effect seen in male cells cultured on SLA and treated with 10^-8 ^M 1α,25(OH)_2_D_3_. Comparison of the mean percent change in response to 1α,25(OH)_2_D_3 _for female and male cells on each of the test substrates showed that, overall, male osteoblasts responded more robustly to the vitamin D metabolite (Table 1).

**Table 1:** Percent increase in response over control in male and female osteoblasts treated with 10^-8^M 1α,25(OH)_2_D_3_.

		% Increase treatment versus control					
		
Sex	Surface	**Cell No**.	Alkaline Phosphatase	Osteocalcin	Osteoprotegerin	Active TGF-β1	Latent TGF-β1
**Male**	TCPS	-4.6	92.5	41.5	185.9	111.8	154.8
	
	PT	-11.3	94.7	37.0	219.9	256.4	162.8
	
	SLA	-13.6	85.0	71.0	224.2	257.8	236.2

**Female**	TCPS	0.3	45.7	17.3	172.1	81.8	60.7
	
	PT	0.2	43.9	20.5	184.8	77.6	98.8
	
	SLA	-3.1	39.4	21.2	141.5	123.3	115.5

TCPS, tissue culture polystyrene; PT, pretreated; SLA, grit-blasted/acid etched.

## Discussion

Despite the large amount of literature on osteoblasts, there has been little assessment of whether these bone forming cells exhibit specific differences related to the sex of the animals from which they were derived. This study examined whether osteoblasts isolated from male and female rats exhibit sexual dimorphism when grown under standard cell culture conditions, as well as when grown on substrates used as implants in clinical practice. In addition, the study addressed the question of whether or not osteoblasts are sensitive to modifications in substrate microarchitecture and if they respond to osteotropic hormones in a sex-specific manner. This study shows that, under control conditions whether on TCPS or smooth or microstructured Ti, there are no apparent differences between cells taken from male or female donors. However, osteoblasts exhibit differences in their responses to oestrogen and in the robustness of their responses to 1α,25(OH)_2_D_3 _that are sex-dependent.

The present study is limited because it was conducted *in vitro *and used rat cells. Thus, the data cannot be correlated directly to human cells or to *in vivo *responses in humans. Here, all of the rats were of the same strain while, in contrast, the human population is outbred and, therefore, inter-human variability may be greater than the differences between the sexes. The goal of this study was to compare the response of male and female osteoblasts grown on implant surfaces. In our population, we have a mixture of osteoprogenitor cells and immature osteoblasts cultured from explants of frontal and parietal bones. Cells with a similar phenotype will migrate to the implant surface *in vivo*, allowing us to mimic the conditions crucial to osseointegration. In order to further this system, we cultured cells only in DMEM and serum and did not include any other factors traditionally used - such as dexamethasone, β-glycerophosphate or ascorbic acid. In order to minimize the potential differences due to cell numbers from individual bone samples, cells were isolated from bones in the same location of the skull, grown in the same media, seeded at the same density and treated at confluence. Thus, in this study the baseline differences between males and females are underscored. We used sexually mature rats for these experiments, so differences in exposure to sex steroids *in vivo *may have affected the subpopulations of osteoblasts isolated from the donor bone.

Even with these limitations, however, the results indicate that there are physiological differences in male and female osteoblasts and sexual dimorphism in the response to bone regulating hormones. This difference is more pronounced when cells are cultured on substrates other than tissue culture polystyrene. Currently, most preclinical studies of therapeutic interventions are performed in only male or only female animal models. Further investigations on sex specific differences should be performed in order to improve our understanding of cellular responses to biomaterials.

Both female and male cells exhibited a more mature phenotype when grown on SLA than when grown on TCPS or PT. This was indicated by the higher levels of osteocalcin and OPG in their conditioned media. Although we had previously shown that male cells and female cells would exhibit a more differentiated phenotype when grown on SLA in comparison to PT [9], we had not directly compared cells from the same species cultured under near identical conditions. The present results remove this ambiguity.

In contrast, the responses of cells cultured on the microstructured surfaces to 1α,25(OH)_2_D_3 _and E_2 _were sex-specific. Male cells were more sensitive to 1α,25(OH)_2_D_3 _than female cells, responding at lower concentrations. Moreover, the response of the male cells to the higher concentration of 1α,25(OH)_2_D_3 _was more robust. In addition, only the female osteoblasts exhibited responses to E_2 _or E_2_-BSA. This confirms our previous results, which showed that osteoblasts from human females exhibit surface-dependent responses to 17β-oestradiol [19], but we had not determined if male osteoblasts would also exhibit a response to E_2 _or E_2_-BSA. Our studies assessing the effects of E_2 _and E_2_-BSA on human articular chondrocytes and rat growth plate chondrocytes demonstrated that male cells lacked a response to 17β-oestradiol and exhibited more robust responses to 1α,25(OH)_2_D_3 _than female chondrocytes [45], suggesting that these are reflective of general properties of cartilage and bone cells. Another possibility is that the male and female rat osteoblasts were at different states of maturation, since response to these systemic regulators varies with the state of the cell within the osteoblast lineage [38].

The mechanisms that mediate these differences are not clear. One possibility is that there are differences in vitamin D receptor (VDR) and oestrogen receptor (ER) expression in female and male cells [30]. Alternatively, components of the signalling pathways involved may play a role. Both 1α,25(OH)_2_D_3 _and 17β-oestradiol act via classical steroid hormone receptors and via membrane-dependent signalling pathways. We did not specifically assess whether there were differences in the membrane signalling by 1α,25(OH)_2_D_3_, but our finding that E_2_-BSA, which cannot enter the cell due to its size [41], had no effect on male osteoblasts indicates that this mechanism of oestrogen action is not operative in cells from male donors. Moreover, E_2 _acted only on female osteoblasts in order to reduce cell number, suggesting that even the traditional ER response was not operational in the male cells. Male rat osteoblasts possess traditional ERs [46], further supporting the hypothesis that the sex-specific effects noted in the present study were mediated by signalling pathways not functional in male cells.

The results suggest that the level of osteotropic hormones such as 1α,25(OH)_2_D_3 _and 17β-oestradiol is important to the regulation of osteoblasts during implant osseointegration. In cases where the hormone level is reduced, such as in postmenopausal females and in vitamin D deficiency [24], the success of the implant may be reduced. In the last decade, an oestrogen effect on the male skeleton was established, although not as extensively as in females. During ageing female bone loss is due to an increased rate of bone resorption, whereas male bone loss is the result of less bone formation [47], indicating that different mechanisms are involved. There is also a sex-related difference in osteocyte lacunar density in human vertebral cancellous bone [48]. Sexual dimorphism may be attributed to differences in ER isoform expression [49] or in the number of receptors [46]. Males with deficiency of ERs or aromatase had defects in skeletal phenotypes [50], indicating that oestrogen is an important regulator for male cells. Alternatively, the higher aromatase activity present in male cells [51] may convert oestrogen to testosterone, thereby reducing the effective concentration of the hormone in the male cells. Whether these mechanisms accounted for the lack of a response to oestrogen noted in the present study is not known.

## Conclusions

The results of this study revealed the physiological difference in male and female osteoblasts and sexual dimorphism in the response to bone regulating hormones, a difference that is more pronounced when cells are cultured on substrates other than tissue culture polystyrene. Currently, most preclinical studies of therapeutic interventions are performed in only male or only female animal models. Further investigations on sex-specific differences should be performed in order to improve our understanding of cellular responses to biomaterials.

## Competing interests

The authors declare that they have no competing interests.

## Authors' contributions

RON designed the study, performed the experiments, analysed the data and helped to draft the manuscript. SLH performed experiments, analysed data and helped to draft the manuscript. RAC and GZ performed the immunoassays. BDB designed the study, analysed data and helped to draft the manuscript. ZS designed the study, analysed data and performed statistical analysis.
